# Do different growth rates of trees cause distinct habitat qualities for saproxylic assemblages?

**DOI:** 10.1007/s00442-021-05061-z

**Published:** 2021-10-17

**Authors:** Kadri Runnel, Jörg G. Stephan, Mats Jonsell, Kadi Kutser, Asko Lõhmus, Joachim Strengbom, Heidi Tamm, Thomas Ranius

**Affiliations:** 1 Department of Ecology, Swedish University of Agricultural Sciences, Box 7044, 75007 Uppsala, Sweden; 2 Institute of Ecology and Earth Sciences, University of Tartu, Vanemuise 46, 51003 Tartu, Estonia; 3 SLU Swedish Species Information Centre, Swedish University of Agricultural Sciences, Box 7007, 75007 Uppsala, Sweden

**Keywords:** Biotic homogenization, Coarse woody debris, Joint species distribution model, Microbiota, Threatened species

## Abstract

In production forests, a common silvicultural objective is enhancing tree growth rates. The growth rate influences both mechanical and biochemical properties of wood, which may have an impact on dead wood inhabiting (i.e. saproxylic) species. In this study, we tested for the first time whether tree growth rates affect dead-wood associated assemblages in general and the occurrence of red-listed species in particular. We sampled saproxylic beetles (eclector traps) and fungi (DNA metabarcoding of wood samples) in dead trunks of Norway spruce (*Picea abies*), which had different growth rates within the same hemiboreal forests in Sweden. A high proportion of fungi showed a positive association to increasing tree growth. This resulted in higher fungal richness in fast-grown trees both at the trunk scale and across multiple studied trunks. Such patterns were not observed for saproxylic beetles. However, a set of species (both beetles and fungi) preferred slow-grown wood. Moreover, the total number of red-listed species was highest in slow-grown trunks. We conclude that dead wood from slow-grown trees hosts relatively fewer saproxylic species, but a part of these may be vulnerable to production forestry. It implies that slow-grown trees should be a target in nature conservation. However, where slow-grown trees are absent, for instance in forests managed for a high biomass production, increasing the volumes of dead wood from fast-grown trees may support many species.

**Supplementary Information:**

The online version contains supplementary material available at 10.1007/s00442-021-05061-z.

## Introduction

Production forestry is globally transforming and homogenizing forest structure, which profoundly affects biodiversity (Noble and Dirzo 1997). A major process across multiple spatial scales is the habitat degradation for most wood-inhabiting (saproxylic) organisms, which has attracted much research (Siitonen 2001; Bunnell and Houde 2010). In boreal forests, it has been estimated that around 25% of all forest organisms are saproxylic (Siitonen 2001). It is well known that distinct saproxylic assemblages inhabit wood of different tree species, dimensions, decay stage, and depending on whether the wood is standing or fallen on the ground (Stokland et al. 2012). Much less known are the consequences of the silviculture for promoting tree growth by thinning, draining, fertilizing, or selective tree breeding. A clear limitation for understanding those effects is a scarcity of basic field research on wood habitat quality in relation to its growth rate. Such research is also relevant for understanding how saproxylic biodiversity could respond to climate-change induced changes in the tree-growth.

Some possible pathways of how tree growth rate can affect wood-inhabiting assemblages are revealed by ecological theory, laboratory experiments and wood technology studies. Live trees have a trade-off between faster growth and structural and chemical defense mechanisms (Loehle 1988). For wood-inhabiting species, this may imply better accessible resource conditions in fast-grown wood, compared to the more stressful conditions in slow-grown wood. Slow-grown wood of many tree species is denser, has thicker cell walls and contains more lignin (Mäkinen et al. 2002; Saranpää 2003; Sarén et al. 2004; Novaes et al. 2010); these properties inhibit the development of decayer assemblages (Stokland et al. 2012). This may end up in a slower decay rate, which may benefit rare species due to an extended time-window for colonization (Edman et al. 2006; Venugopal et al. 2016). Furthermore, laboratory experiments show that some decayer fungi are more efficient in degrading slow-grown conifer wood, and may have a competitive advantage there (Edman et al. 2006; Venugopal et al. 2016). Additionally, tree growth rate can affect wood-inhabiting assemblages through the combined influences of cambium growth and morphology to produce specific bark structure of slow-grown trees (Whitmore 1963; MacFarlane and Luo 2009; Villari et al. 2014). This structure may affect many invertebrates inhabiting the cambium and bark. Indeed, casual observations suggest an association of several saproxylic beetle species with slow-grown trees (Ehnström 2001; Ehnström and Axelsson 2002), but this has never been tested.

Ecological field studies have not explicitly analyzed the tree-growth effects on saproxylic assemblages. Instead, these effects are usually integrated with other factors. Perhaps the best documented case is the distinct lichen and fungal assemblages in old decorticate Scots pines (*Pinus sylvestris*), called ‘kelo’ trees in North Europe (Niemelä et al. 2002; Santaniello et al. 2017). However, the studies on ‘kelos’ have so far not distinguished the tree growth effects from exposure time and habitat. Similarly, some stand-scale studies have indicated relatively high biodiversity values in unproductive pine forests on rocky outcrops (Hämäläinen et al. 2020; Jönsson and Snäll 2020) where, again, possible tree-growth effects are combined with multiple other substrate and stand-history factors. Finally, the tree-scale studies that correlate wood densities with biodiversity measures, even if restricted to one decay stage of the same tree species (e.g. Janssen et al. 2011), are probably confounded by the advancement of decay to allow inference on the specific effect of tree growth rate.

In this study, we test the role of tree growth rate in structuring the assemblages of saproxylic fungi and beetles. Fungi and beetles constitute the two largest groups of saproxylic species, with a large fraction of species now threatened in intensively managed forest regions (Stokland et al. 2012). To explicitly investigate the tree-growth effect, our approach is to sample dead trees of the same dimensions but with contrasting growth rates in the same forests. The wood structure of the study species, Norway spruce (*Picea abies*), is known to be much affected by its growth rate (Saranpää 2003). We expect that dead wood of slow-grown trees: (1) is less species-rich due to a more stressful micro-environment, but (2) may host species specifically associated with this habitat. We test these hypotheses at the trunk scale, and for multiple studied trunks to understand species accumulation due to variation among trunks.

## Materials and methods

### Study sites and field measurements

**Fig. 1:**
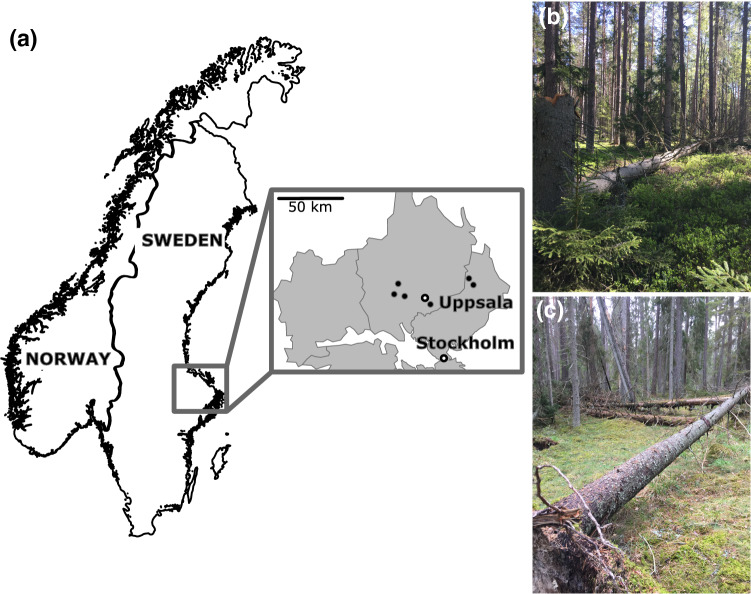
**a** Map of Scandinavia and locations of the six study sites in the counties of Uppsala and Stockholm in Sweden. Photos illustrate **b** a fast growing tree on fertile soil and **c** the corresponding slowly grown tree on a higher rocky part in Lunsen forest reserve

We performed the study in spring–autumn 2019 in six nature reserves (Anddalsglupen, Fiby, Lunsen, Pansarudden, Styggkärret, Svanhusskogen; hereafter: the study sites) in central Sweden (Fig. 1), in the hemiboreal vegetation zone (Ahti et al. 1968). The study sites were rocky areas, 87–398 ha in size. All were dominated by > 100 year-old forests on rather flat terrain below 200 m a.s.l., surrounded by farmland and commercially managed conifer forests. Norway spruce and Scots pine were codominant tree species in all study sites.

The sampling included both standing and fallen spruce trunks, which are known to host partly different saproxylic assemblages (e.g. Lindhe et al. 2004). In each study site, we aimed to sample 16 trunks (8 standing and 8 fallen) divided equally between slow- and fast-grown trees found in the local conditions. Slow-grown trees were usually found in the rockiest parts of the study sites, while fast grown trees were often found near the reserve borders adjacent to production forests. The trunks were at a distance of at least 50 m from each other. The trunks qualified for sampling if they were: (1) growing on mineral soils on dry forest land, (2) 15–35 cm in diameter (fallen trunks—at the sampled location; standing trees—at breast height), (3) covered by bark by at least 90%; (4) in early to medium decay stage (Renvall 1995). The wood of the early decay stage trunks was still hard; the medium decay wood could be penetrated by knife easily for 1–3 cm. The rationale for such a decay stage criterion was to capture both characteristic beetle and fungal assemblages: early decay stages are known to host beetle assemblages that are specifically related to the host tree (Jonsell et al. 1998), whereas many threatened fungi with specialized habitat requirements are associated with medium and later decay stages (Runnel et al. 2021). In one site, we only found six suitable standing trees; hence, the total sample size was 94 (48 fallen and 46 standing trees).

Tree growth rate (average diameter increment mm year^−1^; hereafter growth rate) was measured from the tree diameter (including bark) and the number of growth rings in an increment core taken at breast height. In 54% of the cases the study trunk was too decayed for coring; the growth rate was then estimated based on an increment core taken from the closest living spruce with similar dimensions and appearance (bark morphology, size, density and angle of branches). The assumption was that such trees had a similar growth rate since the growth conditions have been similar. We checked this assumption by measuring fourteen pairs of study trunks and their closest same-sized living trees, which revealed a reasonably close correlation between their growth rates in our study system (*r*^2^ = 0.79; ESM Appendix 1).

We also estimated the light conditions of each study trunk, as these are known to have an effect on saproxylic assemblages (e.g. Rayner and Boddy 1988; Seibold et al. 2016). Hemispherical canopy (fisheye) photos were taken in August, when tree foliage was fully expanded. For fallen trees, they were taken from the center of the sampled location and for standing trees, by combining two photos taken at the breast height from two sides of the tree. The grain size was kept constant, and canopy openness (proportion of sky visible) was calculated using Gap Light Analyzer (Frazer et al. 1999), using blue color plane to improve color contrast between sky and non-sky.

### Beetle and fungal data

Beetles living in each study trunk were collected, using eclector traps (an improved version of Alinvi et al. 2007) covering a standard-sized surface area (around 0.5 m^2^). These traps represent a non-destructive sampling method, where all beetles that emerge from the trunk section (both bark and wood) are captured into a collecting recipient (Alinvi et al. 2007). Using a standard-sized sampling area enables direct comparison of species richness and composition among trees. The traps were in the field from April to August 2019 (the emerging period of most saproxylic beetles) and were emptied once, at the end of sampling period. Five traps were found damaged, and were excluded from the study. Hence, for beetles the total number of analyzed trunks was 89 (43 fallen and 46 standing). The beetles were identified to species following Lundberg and Gustafsson (1995). Only saproxylic species were analyzed, while the random occurrences of herbivorous species from the forest vegetation were omitted. The saproxylic species and their functional (feeding) guilds were delineated based on Palm (1959), Hansen (1964), and Koch (1992).

Wood (sawdust) samples for high-throughput sequencing of fungal DNA were collected in April 2019. The method was adapted from Runnel et al. (2015): five drilling holes were made with a sterilized cordless drill (bit diameter 10 mm) alternately on opposite sides of each trunk. In fallen trunks, the holes were drilled along a 4 m-long sections (1 m intervals) at least 1 m from the tree base. In standing trees, the lowest and highest holes were drilled at 30 cm and 1.8 m height, respectively, and the three remaining holes were distributed between these. Before drilling, the bark and slim wood layer were removed from the drilling point with a knife (which was ethanol flamed to avoid cross contamination). The five wood samples per trunk were pooled, and the drill bit was ethanol flamed between sampling different trunks. The samples were air dried at room temperature for ca. 24 h.

The DNA-metabarcoding analyses and bioinformatics of the fungal data follow Tedersoo et al. (2014). The workflow for the molecular analyses included: (1) DNA extraction using DNeasy PowerSoil DNA Isolation Kit (Qiagen GmbH, Hilden, Germany); (2) PCR for amplifying rDNA ITS2 marker using primers gITS7ngs and ITS4ngsUni (Tedersoo and Lindahl 2016); (3) library preparation and Illumina MiSeq sequencing using 2 × 250 bp paired-end mode at the Institute of Genomics (University of Tartu, Estonia). The following steps in the bioinformatics were: (4) quality filtering, trimming and clustering using programs implemented in PipeCraft 1.0 (Anslan et al. 2017). The clustering was done using the UPARSE operational taxonomic unit (hereafter: OTU) algorithm (Edgar 2013) with a 97% similarity threshold; (5) taxonomic assignment of OTUs using BLASTn analyses against INSDC (International Nucleotide Sequence Databases Collaboration) and UNITE 8.0 (UNITE Community 2019) databases, and removal of non-fungi and OTUs with poor BLASTn values; (6) assignment of OTUs to functional guilds based on FUNGuild (Nguyen et al. 2016). A detailed technical overview of the molecular analyses and bioinformatics is given in ESM Appendix 2.

To better assess conservation relevance, red-listed species were distinguished both in the beetle and fungal datasets. These were defined as either threatened or Near Threatened taxa in any of Fennoscandian Red Lists since 2000 (e.g. Gärdenfors 2000; Henriksen et al. 2015; Artdatabanken 2020). The rationale for such definition is that in the latest red-list versions almost no species have been added, while many species have been excluded because of a stricter interpretation of the red-listing criteria after 2000. However, most excluded species nevertheless indicate sensitivity to anthropogenic change and are considered of conservation value (Nitare 2019).

### Data processing

We assessed the effect of tree growth rate on the assemblages of saproxylic beetles and fungi separately at the trunk scale and for multiple studied trunks. We also addressed separately frequent, infrequent, and red-listed species in the dataset.

(i) To test whether three growth rate influences species/OTU accumulation across multiple studied trunks, sample-based species rarefaction and extrapolation curves (Colwell et al. 2012) were constructed based on presence-absence matrix per study trunk. The trunks were divided into three equal groups based on ordered growth rate values. To mitigate the confounding effect of trunk type, the three groups were formed separately for fallen and standing trunks and then pooled. Separate rarefaction curves were calculated for beetles, fungi, and all red-listed species (fungi and beetles pooled). We used raw incidence data and the command iNEXT from the INEXT package (Hsieh et al. 2016).
(ii) Detailed trunk-scale effects were analyzed using Hierarchical Modeling of Species Communities (HMSC; Ovaskainen and Abrego 2020). HMSC is a joint species distribution modelling approach that estimates the occurrences and/or abundances of each species based on environmental covariates and species co-occurrence patterns using the matrix of species occurrences by sampling units (trunk) as response. Results for individual species, several species (e.g. different guilds), or the whole assemblage can be extracted. As each species’ realized niche is modelled, this approach could be used only for species with at least five occurrences (hereafter: frequent species). The models were fitted separately to fungi and beetle data (ESM Appendix 3), using the R-package Hmsc (Tikhonov et al. 2020). For fungi, we used a hurdle model, in which we first fitted a model for OTU presence-absence data (Bernoulli distribution with probit link function), and then another model for OTU abundances (sequence-counts) conditional on presence (normal distribution with identity link function). For beetles, we only modelled presence-absence as the biological relevance was mostly captured by the fact that a species was found or not.
  In addition to the tree growth rate (continuous predictor), the fixed factors in the HMSC models included “trunk diameter” and “canopy openness” (both continuous), and the categorical variables “trunk type” (two levels: fallen and standing) and “decay stage” (two levels: “early” and “medium”). The continuous fixed factors were not correlated (Pearson correlations *r* < 0.22 for all combinations). To account for the hierarchical structure of the study design (trees nested within study sites), we included the study site as a random effect, meaning the model could also use the co-occurrence at this level. In the models for fungi, “total number of sequences” (a log transformed continuous variable) was included to control for variation in sequencing depth. The explanatory power of the models was assessed using *R*^2^ (Tjur’s *R*^2^ in binomial models). Tjur’s *R*^2^ is the coefficient of discrimination for generalised linear (mixed) models for binary outcomes (presence-absence) (Tjur 2009). MCMC chain convergence was evaluated quantitatively by estimating effective sample sizes and potential scale-reduction factors (Gelman et al. 2014) (ESM Appendix 3). To explore the effect of tree growth rate on individual species, we examined the *β*‐parameters (regression slopes of occurrence probability/abundance depending on growth rate). To explore the effect of tree growth on ecologically distinct sets of species (saprotrophs vs others in fungi; fungivores, wood and cambium consumers in beetles) we summarized the predictions of richness along growth rate for these functional guilds.
(iii) To examine the response of those species/OTUs that occurred in fewer than five trunks (infrequent species), we performed an analysis of their trunk-scale species richness. We used linear mixed-effects models in R-package lme4 (Bates et al. 2014). For fungi, we assumed a negative binomial error distribution (log link function), and for beetles a Poisson error distribution (log link function). The same fixed and random effects were used as for the HMSC analyses, but we also tested for the “growth rate” × “decay stage” interaction. The latter addressed the question whether the putative growth rate association might be stronger at the beginning of the decay (for pioneer colonizers), given that chemical and physical wood properties become less specific along the decay process. Since such effect was not significant, it was omitted from the final models. We standardized the continuous predictor variables prior to analysis, and assessed adequate residual distribution using the DHARMa package (Hartig 2018). Marginal and Conditional *R*^2^ (package MuMIn; Barton and Barton 2019) were used for evaluating model fits. The former represents the proportion of total variance explained through fixed effects only, the latter combines both fixed and random effects.

## Results

The tree growth rate across all studied trunks ranged from 1.3 to 8.9 mm year^−1^ (mean 3.7; SD ± 1.60). The captured growth rate range differed among study sites; within study sites, the trees differed in their growth rate by 4.0–7.5 mm year^−1^. The average growth rate was slightly larger in fallen trunks (3.9; SD ± 1.7) than standing trunks (3.4; SD ± 1.4), but this difference was not statistically significant (Linear regression; *t*(standing trunks) = − 1.30; *p* = 0.199). When the sample was divided into three equal parts according to the growth rate, the fast-grown trees had a wider range (3.9–8.9 mm year^−1^) than the slow-grown (1.3–2.7 mm year^−1^) or medium trees (2.7–3.9 mm year^−1^).

In total, we recorded 121 saproxylic beetle species (22,750 species records) in the studied trunks. After quality filtering, the final dataset for fungi comprised 2064 OTUs (5,445,958 sequences). Both assemblages were dominated by rare taxa: 38% of beetle species and 57% of fungal OTUs occurred in only one trunk. Among beetles the prevailing functional guild was fungivores (28%). Among fungi, the functional guilds could be assigned to 37% of the OTUs and the prevailing guild was saprotrophs (63.3%).

**Fig. 2:**
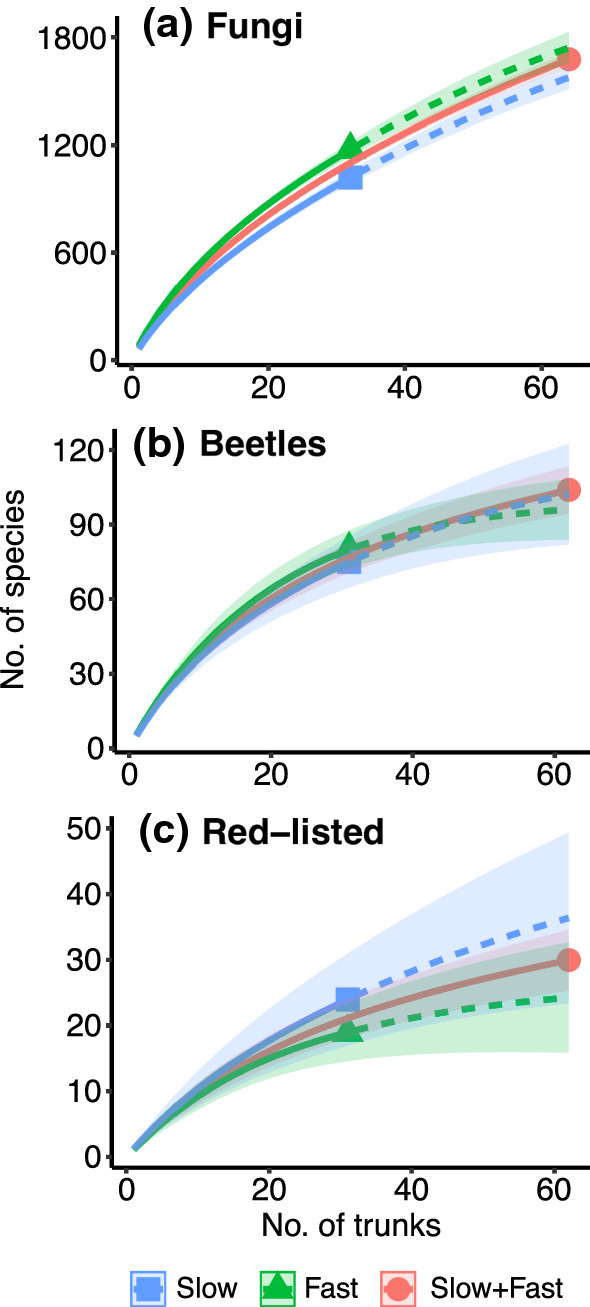
Species accumulation curves for **a** fungal OTUs, **b** beetle species, and **c** red-listed species (beetles and fungi pooled) in the trunks with the highest and lowest tree growth rate. The red line depicts rarefaction for the two growth rate groups pooled. For the sake of clarity, the overlapping case of the trunks with a medium growth rate are not shown. Solid line is the interpolated rarefaction curve, dashed line is the extrapolated curve for double sample size; shaded regions are 95% confidence intervals

The fastest grown trees (first third) hosted a larger total number of fungal OTUs than the slowest grown ones (last third) (Fig. 2a). Their pooled curve did not exceed that of the fastest grown trees, suggesting that the OTUs detected in the slowest grown group were largely a subset of the former (Fig. 2a). In the case of beetles, the species accumulated similarly on trees of all growth rates (Fig. 2b).

Thirty beetle species and 334 fungal OTUs occurred in at least five trunks and were included in the joint species distribution models (HMSC). About 10% of the explained variation in the models could be attributed to the tree growth rate in both beetles and fungi (Table 1). In both groups, the major factor explaining 30–32% of the assemblage composition was the decay stage (Table 1). In beetles, the fallen/standing contrast added 23% of the explained variation (standing trunks preferred).

**Table 1:** Community-level summary of the HMSC models assessing environmental factors for frequent fungal OTUs and beetle species in spruce trunks

	Fungi						Beetles		
	Probability of presence (*R*^2^ Tjur = 0.12)			Abundance conditional on presence (*R*^2^ = 0.63)			Probability of presence (*R*^2^ Tjur = 0.08)		
	Explained variance (%)	Mean diff.	*p*[effect > 0] (%)	Explained variance (%)	Mean diff.	*p*[effect > 0] (%)	Explained variance (%)	Mean diff.	*p*[effect > 0] (%)
Early/late decay	30.3	6.29	100	11.9	182.73	100	32.1	− 0.54	85.7
Tree growth rate	9.7	10.07	99.9	13.0	405.58	100	11.7	− 0.27	61.7
Canopy openness	10.4	− 20.29	100	14.3	263.48	100	13.1	2.36	99.2
Standing/fallen	8.6	3.66	99.2	13.9	72.88	95.6	23.4	− 0.83	95.5
Trunk diameter	9.0	3.66	93.3	13.8	− 37.30	74.1	10.6	0.21	60.9
Sequencing depth	17.9	36.28	100	23.3	556.13	100			
Random: study site	14.0			9.8			9.1		

Mean diff. shows the difference in species/OTU richness between the levels of categorical variables or between the minimum and maximum of continuous variables (summed posterior means of each species response to the variable). For decay stage (two levels: early/late), early stage was used as reference; for trunk type (two levels: standing/fallen), fallen trunk was the reference. *p*[effect > 0] shows the probability of an effect (proportion of posterior distributions of the difference above zero)

A larger number of fungal OTUs included in the HMSC analyses had a positive than a negative response to the tree growth rate. For example, at the level of support of 75% posterior probability, there were 45 OTUs with a negative response and 75 with a positive response; this resulted in a positive assemblage-level effect of tree growth rate on species richness (probability of presence model: *p*[effect > 0] = 99.9%; Table 1). The latter pattern was also evident for a subset of fungal OTUs that could be reliably ascribed to the guild of saprotrophs (ESM Appendix 4). The outcome of the model for fungal abundance conditional of presence largely mirrored that of the presence-absence model. However, in this model the importance of the decay stage in explaining the variation decreased, while that of other factors increased. For beetles, the 75% posterior probability distinguished six species with a negative and five species with a positive response; their assemblage-level growth rate effect was neither significant overall (*p*[effect > 0] = 61.7%; Table 1) nor by functional guilds (ESM Appendix 4).

The number of infrequent fungal OTUs per trunk (*n* = 1730) was not significantly related to any measured environmental factor (Table 2). The number of such OTUs per trunk also fluctuated more than that of frequent fungal OTUs (coefficients of variation 106% vs 66%, accordingly). The species richness of infrequent beetle species (*n* = 91) tended to be higher in fallen than standing trunks and early than late decay stages, and it also increased with the trunk diameter.

**Table 2:** Results of GLMMs explaining the numbers of infrequent fungal OTUs and beetle species in spruce trunks

	Fungi (*R*^2^ = 0.19/0.21)			Beetles (*R*^2^ = 0.14/0.18)		
	Estimate	*z*-value	*p*	Estimate	*z*-value	*p*
Early/late decay	0.26	1.49	0.138	− 0.30	− 1.91	0.055
Tree growth rate	0.07	0.69	0.492	0.03	0.37	0.711
Canopy openness	0.02	0.23	0.816	− 0.06	− 0.63	0.527
Standing/fallen	0.01	0.07	0.945	− 0.33	− 1.87	0.062
Trunk diameter	0.01	0.10	0.922	0.25	2.90	0.004**
Sequencing depth	0.34	3.60	< 0.001***			

For decay stage (two levels: early/late), early stage was used as reference; for trunk type (two levels: standing/fallen), fallen trunks were a reference. Marginal/conditional *R*^2^ given for the whole model

Asterisks indicate *p*-values as follows: ***p ≤ 0.001, **0.001 < *p* ≤ 0.01, *0.01 < *p* ≤ 0.05,∙0.05 < *p* ≤ 0.1

We detected in total 35 red-listed species (23 beetles and 12 fungi). The number of such species (beetles and fungi together) accumulated faster among the slowest grown trunks, but the differences from fast-grown trunks remained small. As pooling the two growth-rate groups did not result in higher overall species numbers, the red-listed species occurring in fast-grown trees rather formed a subset of those occurring in slow-grown trunks (Fig. 2c). The response was explicitly explored in seven red-listed beetle and one fungus species that were abundant enough to be included in the HMSC models (ESM Appendix 5). Among these species, the tree growth rate had a distinguishable influence on the fungus *Phellinus ferrugineofuscus*: its presence did not significantly respond to tree growth rate, but its abundance conditional on presence was largest in slow-grown trunks.

## Discussion

Our study provides partial support to the expected pattern that slow-grown trees have fewer species, but a part of these are specialists and potentially vulnerable to production forestry.

We found an overall increase in species richness of fungi, but not of beetles, in response to increasing tree growth rate. This probably reflects a true difference between these organism groups. It is less likely that this result was due to the smaller representation of the saproxylic beetle diversity in our samples, since the eclector trap data of beetles is rather accurate (the traps capture only those beetles that actually live in the tree). Fungal meta-barcoding data, on the other hand, may reveal also non-functional organisms (e.g. dead or otherwise random or non-viable fungal occurrences) (Carini et al. 2016; Tuovinen et al. 2015). Moreover, the variances explained by the tree growth rate in the species distribution models were similar in the fungal and beetle analyses, both in absolute terms and relative to other major variables (Table 1).

To clarify the observed positive relationships between tree growth rate and fungal species richness, we first explain a seemingly contradictory result. We detected such assemblage level response (1) on the rarefaction curves for the whole species pool, and (2) in the species distribution models for frequent species, but not (3) in the species richness models of infrequent OTUs. The latter contributed with 84% of all fungal OTUs recorded, so it is not obvious why the rarefaction indicated a significantly larger overall species pool for rapidly grown trees (Fig. 2a). We suspect a technical explanation: the occurrence of rarer fungi (on average, 40% of OTUs at the trunk scale) fluctuated too widely for detecting an effect of growth rate on their per trunk richness. This is supported by the facts that the measured variation was much higher in infrequent than frequent fungal OTUs, and that the joint species distribution models revealed also the well-known effects of decay stage, trunk type, and canopy openness (e.g. Lonsdale et al. 2008; Rajala et al. 2012), while in the analyses of the infrequent OTUs those effects remained undetected. Thus the result for the frequent fungi—that a positive tree growth rate effect for OTU richness arises because a higher number of OTUs benefit from faster than slower tree growth—may be valid for the whole fungal pool.

One could expect that the observed larger species pool of fungi in rapidly grown wood is due to species with generalist, competitor or ruderal characteristics. This is because, as explained above, opportunistic consumption of rapidly grown wood is supported by its weaker mechanical and chemical resistance. Some species-level effects detected in our fungal species models support this idea. For example, among the well-known group of polypore fungi, positive tree growth-rate influences were detected for the widespread parasite *Heterobasidion parviporum*, and for *Trichaptum abietinum*—a saprotroph with ruderal traits (cf. Pasanen et al. 2014). Similarly, Edman et al. (2006) reported fast-grown wood providing an advantage to a generalist over a specialist species in the genus *Fomitopsis*. A detailed analysis of fungi at the functional-guild or life-history trait level was unfortunately not possible in our dataset, because a large proportion of OTUs remained identified on above-species taxonomic level only (common in metabarcoding datasets). Thus, we cannot reject an alternative hypothesis: that the generally more favorable fast grown substrate supports a larger total number of fungal colonizations, which leads to a larger number of species as a probabilistic result.

We also found that slow-grown trees tended to support more red-listed species (beetles and fungi combined). Most forest species currently red-listed in North Europe are habitat specialists that have small and fragmented or declining populations (Tikkanen et al. 2006; Nordén et al. 2013). Our result suggests that silvicultural treatments that accelerate and homogenize tree growth across forest landscapes might threaten red-listed species. In our dataset, this was confirmed for the redlisted polypore *Phellinus ferrugineofuscus*, which was found in trunks with different growth rates, but attained larger abundance (once present) in slow-grown trunks. This species is known to become rare in intensively managed spruce forests (Peltoniemi et al. 2013) and it has not been reported to inhabit chainsawed logs where it is probably outcompeted by species with ruderal traits (Komonen et al. 2014; Pasanen et al. 2014). We recognize that this relationship was documented based on only six records, and requires further study. However, *Phellinus ferrugineofuscus* could potentially serve as a focal species that indicates the condition for biodiversity related to slow-grown dead wood in managed forest landscapes (Lõhmus et al. 2020).

Our study was not designed to document effects of extreme wood growth rates because such extremes tend to occur in otherwise different conditions. Rather, we addressed the variation in tree growth rates within the limited set of conditions in a certain forest type in one region in order to explore its independent contribution. The results provide good justification for further studies in other ecosystems.

## Implications for nature conservation

In practical terms, two broad implications can be drawn from our study. First, since our results support the view that a fraction of species mainly occurs in slow-grown trees, such trees should be a conservation target. In boreal forests, that may be of even higher importance in the future, since forest management and climate change are expected to increase tree growth rates (Weslien et al. 2009) and, hence, a decreasing trend for slow-grown trees can be expected. The current knowledge does not allow identifying any ‘natural baseline’ for the abundance of slow-grown trees at any scale. Yet, studies in old-growth forests indicate that slow-grown trees are relatively abundant, i.e., the growth-rate distributions are positively skewed there (Kohyama and Hara 1989; Finegan et al. 1999). In contrast, tree growth is artificially accelerated in production forests by regular removal of suppressed trees. Therefore, old forests can be important for maintaining such trees on the landscape scale. Also low-productivity forests not used for forestry might be important for certain species (e.g. those utilizing Scots pines in wooded mires or rocky outcrops; Hämäläinen et al. 2018, 2020). In production forests, the techniques for maintaining slow-grown trees include retaining existing trees at harvests (‘retention forestry’; Gustafsson et al. 2012) and tolerating some tree suppression locally (e.g. as multi-layered stands or by sparing parts of thickets from thinnings).

Our second implication regards the restoration of dead-wood pools in biologically impoverished production landscapes (reviewed by Sandström et al. (2019)). In such landscapes, slow-grown trees may be rare, and their re-establishment may take decades, if it is possible at all. However, our study indicates that rapidly-grown trees provide both abundant and well accessible resource for most saproxylic species, and the trunks host larger total numbers of species per volume unit than those of slow-grown trees. Hence, in such landscapes, increasing the amount of dead wood from existing fast-grown trees (both passively by natural mortality, or by actively cutting trees; see also Pasanen et al. 2014; Elo et al. 2019) can still support many species despite missing some species associated with slow-grown wood. However, a long term restoration of such landscapes should also involve an increase in slow-grown trees.

## Supplementary Information

Below is the link to the electronic supplementary material.

Supplementary file1 (PDF 429 KB)

## Declarations

### Availability of data and materials

Data available via the Plutof Platform: 10.15156/BIO/2483891.

### Code availability

The code produced for analyses conducted in this study is available from the corresponding author upon request.
