# Supplementary material for: Do different growth rates of trees cause distinct habitat qualities for saproxylic assemblages?
Source: Oecologia. 2021 Oct 17;197(3):807–16. doi: 10.1007/s00442-021-05061-z (PMC8585823; doi:10.1007/s00442-021-05061-z)
Supplement: Supplementary file 1 — Supplementary file1 (PDF 429 KB) [file 442_2021_5061_MOESM1_ESM.pdf]

Electronic supplementary material

Do different growth rates of trees cause distinct habitat qualities for saproxylic assemblages?

Kadri Runnel, Jörg G. Stephan, Mats Jonsell, Kadi Kutser, Asko Lõhmus, Joachim Strengbom, Heidi Tamm, Thomas Ranius

Appendix 1

The relationship (Pearson correlation) between the growth rate of study trunks (y-axis) and closest living spruce with similar dimensions and appearance (x axis).

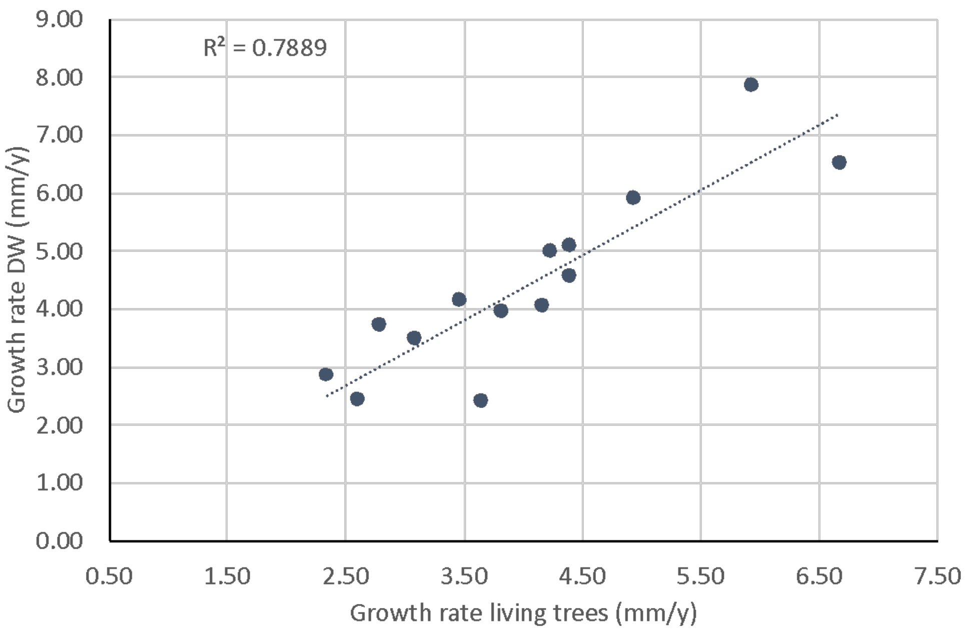

Appendix 2

The protocol of molecular analyses and bioinformatics for sampling fungi from wood

Dried sawdust samples of 100 mg were homogenised using stainless steel beads (Ø 3.2 mm) in a Retsch MM400 homogenizer (Retsch GmbH, Haan, Germany). DNA was extracted with DNeasy PowerSoil DNA Isolation Kit (Qiagen GmbH, Hilden, Germany) following manufacturer's instructions. We selected rDNA ITS2 region for fungal species identification. Primers gITS7ngs and ITS4ngsUni (Tedersoo and Lindahl 2016) were tagged with 12-base identifier barcodes as described in Tedersoo et al. (2014). The PCR mixture comprised 1 µl DNA, 1 µl of tagged gITS7ngs primer (20 µM), 0.5 µl of tagged ITS4ngsUni primer (20 µM), 5 µl 5x HOT FIREPol Blend Master Mix (Solis Biodyne, Tartu, Estonia) and 17.5 µl mQ water. PCR was carried out in duplicate replicates in the following thermocycling conditions: an initial 15 min at 95 °C, followed by 30 cycles of 95 °C for 30 s, 52 °C for 30 s, 72 °C for 1 min, and a final cycle of 10 min at 72 °C. PCR products from replicate samples were pooled and their relative quantity was estimated on 1% agarose gel. DNA samples with low or no visible bands were re-amplified using 35 cycles and 2 µl of DNA template. Both negative (mQ water) and positive controls were included in PCR and sequencing runs. PCR products were pooled at approximately equimolar ratios as determined by gel band strength, with the exception of negative control from which 5 µl were added. The library was purified by FavorPrep™ Gel/PCR Purification Kit (Favorgen Biotech Corp., Vienna, Austria), following the manufacturer's instructions. Thereafter, the library was subjected to ligation of Illumina adaptors using the TruSeq DNA PCR-free Library Prep kit (Illumina Inc., San Diego, CA, USA) and Illumina MiSeq sequencing using 2 x 250 bp paired-end mode at the Institute of Genomics (University of Tartu, Estonia).

Illumina sequencing provided 10,190,731 raw reads that were processed using PipeCraft 1.0 platform (Anslan et al., 2017). Paired-end reads were merged and quality trimmed using vsearch v1.11.1 (Rognes et al., 2016; trimming options: maxee=1, maxee_rate=999, truncqual=5). The resulting 8,145,716 sequences were re-assigned to samples based on the identifier barcodes using mothur v1.36.1 (Schloss et al., 2009; demultiplexing options: bdiffs=1, pdiffs=2). A total of 7,333,412 sequences were subjected to *de novo* as well as reference database (UCHIME v7.1; Nilsson et al. 2015) based chimera filtering using vsearch. To extract the full-length ITS2 subregion the sequences were processed with ITSx 1.0.11 (Bengtsson-Palme et al., 2013) to remove flanking gene fragments. Full-length ITS2

reads were assigned to operational taxonomic units (OTUs) by clustering with USEARCH v8.1.1861 (Edgar 2010) using UPARSE OTU algorithm (Edgar 2013) at 97% similarity threshold. The most abundant sequence of each cluster was selected as a representative for BLASTn sequence similarity search (Camacho et al. 2009) against both INSDC (International Nucleotide Sequence Databases Collaboration) and UNITE 8.0 (UNITE Community 2019). BLASTn e-values < e^{-50} were considered reliable to assign OTUs to kingdoms, whereas OTUs with e-values > e^{-20} were treated as unknown taxa. E-values between e^{-50} and e^{-20} were checked manually against the ten best matches for accurate assignment. The OTUs with e>-20 were removed, as well as all sequences representing non-fungi, and all singletons (OTUs present only as a single sequence read per sample). We relied on 98%, 90%, 85%, 80%, and 75% sequence identity as a criterion for assigning OTUs to species, genus, family, order or class level, respectively (Tedersoo et al., 2014). Each fungal genus, family or order was assigned to functional categories based on FUNGuild (Nguyen et al., 2016).

References

Anslan S, Bahram M, Hiiesalu I, Tedersoo L. 2017. PipeCraft: flexible opensource toolkit for bioinformatics analysis of custom high-throughput amplicon sequencing data. Mol. Ecol. Resour. doi: 10.1111/1755- 0998.12692.

Bengtsson-Palme, J., Ryberg, M., Hartmann, M., Branco, S., Wang, Z., Godhe, A., De Wit, P., Sánchez-García, M., Ebersberger, I., de Sousa, F., Amend, A., Jumpponen, A., Unterseher, M., Kristiansson, E., Abarenkov, K., Bertrand, Y.J.K., Sanli, K., Eriksson, K.M., Vik, U., Veldre, V. and Nilsson, R.H. (2013), Improved software detection and extraction of ITS1 and ITS2 from ribosomal ITS sequences of fungi and other eukaryotes for analysis of environmental sequencing data. Methods Ecol Evol, 4: 914-919. doi:10.1111/2041-210X.12073

Camacho, C., Coulouris, G., Avagyan, V. et al. BLAST+: architecture and applications. BMC Bioinformatics 10, 421 (2009). https://doi.org/10.1186/1471-2105-10-421

Edgar R.C. (2010), Search and clustering orders of magnitude faster than BLAST, Bioinformatics 26(19) 2460-2461

Edgar, R.C. (2013) UPARSE: Highly accurate OTU sequences from microbial amplicon reads, Nature Methods [Pubmed:23955772, dx.doi.org/10.1038/nmeth.2604].

Nguyen, N.H., Song, Z., Bates, S.T., Branco, S., Tedersoo, L., Menke, J., Schilling, J.S., Kennedy, P.G., 2016. FUNGuild: an open annotation tool for parsing fungal community datasets by ecological guild. Fungal Ecology 20, 241–248. https://doi.org/10.1016/J.FUNECO.2015.06.006.

Nilsson RH, Tedersoo L, Ryberg M, Kristiansson E, Hartmann M, Unterseher M, et al. 2015. A comprehensive, automatically updated fungal ITS sequence dataset for reference-based chimera control in environmental sequencing efforts. Microbes Environ. 30: 145-150

Rognes, T., Flouri, T., Nichols, B., Quince, C., Mahé, F., 2016. VSEARCH: a versatile open source tool for metagenomics. PeerJ 4:e2584;, doi: 10.7717/peerj.2584

Schloss PD, Westcott SL, Ryabin T, Hall JR, Hartmann M, Hollister EB, et al. 2009. Introducing mothur: open-source, platform-independent, community-supported software for describing and comparing microbial communities. Appl Environ Microbiol. 75:7537. doi: 10.1128/AEM.01541-09.

Tedersoo, L. & Lindahl, B. Fungal identification biases in microbiome projects. Env. Microbiol. Rep. 8, 774–779 (2016).

Tedersoo, L., Bahram, M., Polme, S., Koljalg, U., Yorou, N.S., Wijesundera, R., Ruiz, L.V., Vasco-Palacios, A.M., Thu, P.Q., Suija, A., Smith, M.E., Sharp, C., Saluveer, E., Saitta, A., Rosas, M., Riit, T., Ratkowsky, D., Pritsch, K., Poldmaa, K., Piepenbring, M., Phosri, C., Peterson, M., Parts, K., Partel, K., Otsing, E., Nouhra, E., Njouonkou, A.L., Nilsson, R.H., Morgado, L.N., Mayor, J., May, T.W., Majuakim, L., Lodge, D.J., Lee, S.S., Larsson, K.-H., Kohout, P., Hosaka, K., Hiiesalu, I., Henkel, T.W., Harend, H., Guo, L. -d., Greslebin, A., Grelet, G., Geml, J., Gates, G., Dunstan, W., Dunk, C., Drenkhan, R., Dearnaley, J., De Kesel, A., Dang, T., Chen, X., Buegger, F., Brearley, F.Q., Bonito, G., Anslan, S., Abell, S., Abarenkov, K., 2014. Global diversity and geography of soil fungi. Science 346. https://doi.org/10.1126/science.1256688.

UNITE Community (2019): UNITE general FASTA release for eukaryotes 2. Version 18.11.2018. UNITE Community. https://doi.org/10.15156/BIO/786354

Appendix 3

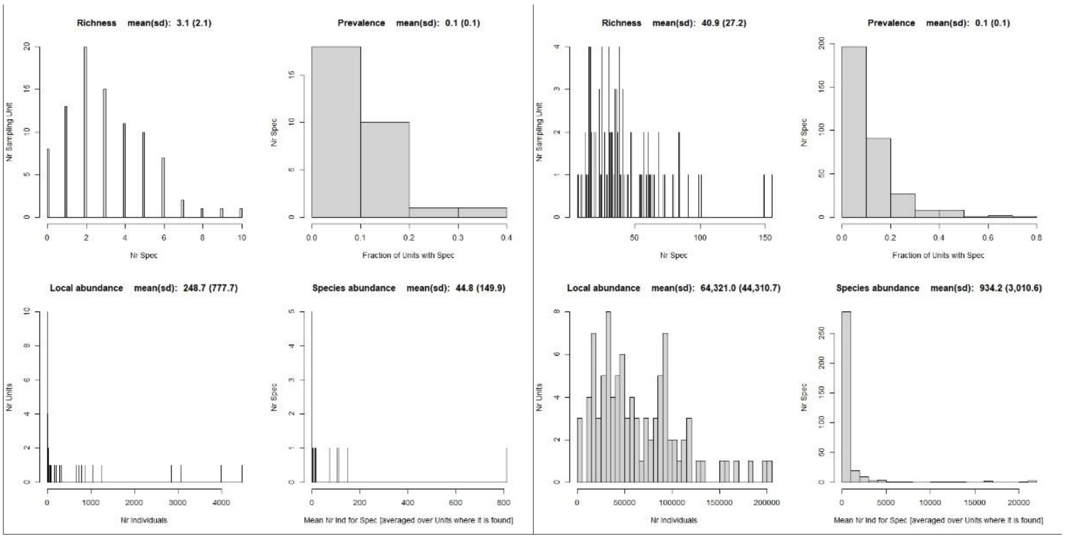

Figure 1. Summary of original data for the beetles (left) and the fungi (right).

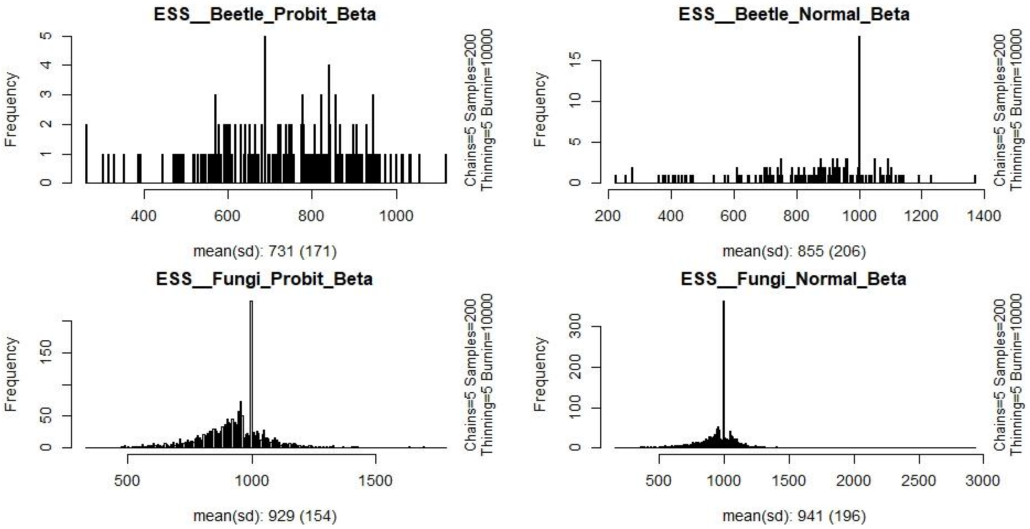

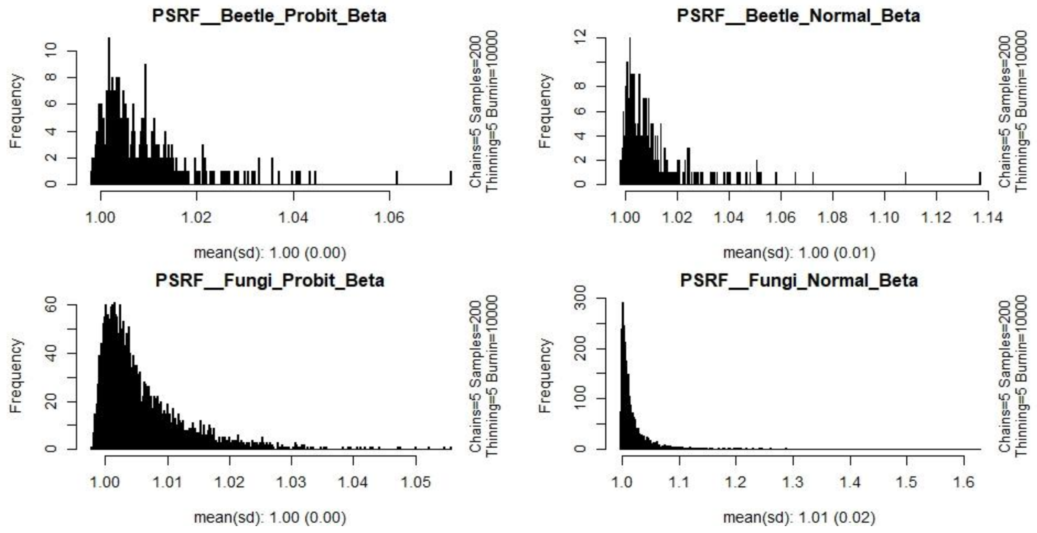

Figure 2. Histograms of model diagnostic for Beta values for each species. Shown are effective sample sizes (ESS) and potential scale reduction factors (PSRF) from gelman diagnostic for each of the four models.

Appendix 4

Community-level summary of the HMSC models assessing environmental factors for functional guilds among frequent fungal OTUs and beetle species in spruce trunks. Mean diff. shows the difference in species/OTU richness between the levels of categorical variables or between the minimum and maximum of continuous variables (summed posterior means of each species response to the variable). For decay stage (two levels: early/late), early stage was used as reference; for trunk type (two levels: standing/fallen), fallen trunk was the reference. P[effect>0] shows the probability of an effect (proportion of posterior distributions of the difference above zero).

	Beetles (presence absence)					
	**Cambium consumers**		**Fungivores**		**Wood consumers**	
	**Mean diff.**	**P[effect>0] (%)**	**Mean diff.**	**P[effect>0] (%)**	**Mean diff.**	**P[effect>0] (%)**
Tree growth rate	0.12	60.5	0.08	56.2	-0.2	70.3
Trunk diameter	-0.16	75.3	-0.01	54.3	0.49	91.3
Canopy openness	1.41	99.8	-0.12	60.6	0.54	91
Early/late decay	0.55	97.9	-0.36	96.2	0.25	87.6
Standing/fallen	0.63	99	-0.29	91.6	0.32	94

	Fungi (presence absence)				Fungi (abund. cond. on presence)			
	**Saprotrophs**		**Others**		**Saprotrophs**		**Others**	
	**Mean diff.**	**P[effect>0] (%)**	**Mean diff.**	**P[effect>0] (%)**	**Mean diff.**	**P[effect>0] (%)**	**Mean diff.**	**P[effect>0] (%)**
Tree growth rate	1.55	89	0.7	83.3	103.66	100	46.73	99.6
Trunk diameter	1.56	89.8	1.25	97.2	-16.24	71.5	24.69	93.4
Canopy openness	-4.76	100	-0.92	87.1	61.09	97.4	41	99.6
Early/late decay	-1.78	99.4	-0.01	52.5	-61.41	99.4	-15.89	90.1
Standing/fallen	-0.98	91.9	0.47	86.9	-30.57	89.7	-0.76	51.9
Sequencing depth	9.74	100	3.67	100	205.73	100	38.29	95.2

Appendix 5

Red-listed species recorded in this study. The **frequent** red-listed species included in the joint species distribution models (HMSC) are given in the table, along with their responses to tree growth rate (slope of the β value) is given. P[effect>0] shows the probability of an increase (proportion of posterior distributions of the difference above zero). For beetles the response variable in HMSC was presence/absence (P), for fungi both presence/absence and abundance conditional on presence (A) were explored. *Phellinus ferrugineofuscus* showed a likely decrease (92 % chance) in occurrence probability with increasing tree growth rate. The **infrequent** red-listed species along with their total no. of occurrences and occurrences per trunk type (Standing/Fallen) are listed below the table.

	No. of occurrences (Standing/Fallen)	Model type	Slope for species response to tree growth rate (median and IQR of posterior distribution)	P[effect>0] (%)
**Beetles**				
Anobium thomsoni	8 (6/2)	P	-0.08 (-0.16, 0.01)	27.7
Atomaria subangulata	6 (2/4)	P	-0.07 (-0.16, 0.01)	26.5
Corticaria lateritia	5 (4/1)	P	-0.06 (-0.15, 0.01)	29.1
Cryptolestes alternans	5 (4/1)	P	-0.00 (-0.10, 0.09)	49.4
Ipidia pinotata	5 (5/0)	P	0.04 (-0.05, 0.12)	61.2
Paromalus flavicornis	5 (2/3)	P	0.03 (-0.06, 0.12)	59.7
Plegaderus vulneratus	12(6/6)	P	-0.08 (-0.17, -0.00)	24.8
**Fungi**				
Phellinus ferrugineofuscus	6 (3/3)	P	0.05 (-0.00, 0.10)	72.7
		A	**-0.17 (-0.25, -0.09)**	**8.3**

Less frequent red-listed species.

Beetles: *Acanthocinus griseus* 4(3/1), *Callidium coriaceum* 4(2/2), *Cis quadridens* 1(1/0), *Corticaria polypori* 1(1/0), *Corticeus fraxini* 1(1/0), *Corticeus suturalis* 4(4/0), *Dendrophagus crenatus* 1(1/0), *Denticollis borealis* 1(0/1), *Euglenes oculatus* 2(1/1), *Microscydmus nanus* 2(2/0), *Orchesia fasciata* 1(0/1), *Phyllodrepa clavigera* 1(0/1), *Ptiliolum caledonicum* 2(0/2), *Scraptia testacea* 1(0/1), *Scydmoraphes minutus* 1(0/1), *Zilora ferruginea* 2(0/2)

Fungi: *Amylocystis lapponica* 1(0/1), *Inonotus leporinus* 2(1/1), *Phellinus viticola* 1(1/0), *Phellopilus nigrolimitatus* 1(0/1), *Postia undosa* 1(1/0), *Pycnoporellus fulgens* 3(2/1), *Sistotrema raduloides* 2(1/1), *Skeletocutis brevispora* 3(1/2), *Skeletocutis kuehneri* 2(1/1)
